# The Update of the Italian Food Composition Database of Gluten-Free Products and Its Application in Food-Based Dietary Guidelines Menus

**DOI:** 10.3390/nu14194171

**Published:** 2022-10-07

**Authors:** Federica Fiori, Maria Parpinel, Federico Morreale, Nicoletta Pellegrini

**Affiliations:** 1 Department of Medicine, University of Udine, 33100 Udine, Italy; 2 Food and Drug Department, University of Parma, 43124 Parma, Italy; 3 Department of Agricultural, Food, Environmental and Animal Sciences, University of Udine, 33100 Udine, Italy

**Keywords:** gluten-free, food composition, food composition database, celiac disease, dietary guidelines, nutritional adequacy, dietary assessment, gluten-free diet, manufactured products

## Abstract

Complete food composition databases (FCDBs) on gluten-free (GF) foods are needed to assess the nutrient intakes of celiac disease patients. The aim of the present work was to update the previously developed version of the Italian GF-FCDB and to apply it to a theoretical GF diet. The updated GF-FCDB includes the composition of 108 GF cereal-based foods, as sold, in terms of energy and macro- and micro-nutrients, imputed using food label information combined with the standard recipe approach. Three scenarios (i.e., refined, mixed, and wholegrain cereals) of the weekly guideline menu for the general Italian population were analyzed for energy and nutrient content in a theoretical dietary assessment using traditional gluten-containing (GC) foods and the corresponding GF substitutes. All GF menus were higher than the corresponding GC menus in polyunsaturated fatty acids, linoleic acid, and vitamin E. Zinc was lower in GF than in GC menus only in the wholegrain-cereal scenario. Thanks to the application of the updated GF-FCDB including a comprehensive list of micronutrients, we observed that it is possible for celiac disease patients to meet nutrient requirements by simply substituting GC with GF cereal-based products following recommendations for the general population.

## 1. Introduction

Celiac disease is a chronic immune-mediated enteropathy occurring in genetically susceptible individuals characterized by a specific serological and histological profile triggered by gluten ingestion [1]. It is one of the most common autoimmune disorders, with a reported global prevalence of 0.7% and 1.4% based on serologic tests and biopsy, respectively, and a high prevalence (0.8%) in European countries [2]. The complete exclusion from the diet of gluten, which is a protein complex present in some cereal products such as wheat, rye, barley, oats, spelt, and their hybridized strains, currently represents the only treatment for celiac disease. The gluten-free (GF) diet is characterized by: naturally occurring GF foods (unprocessed meat and fish, dairy products, eggs, legumes, fruit, and vegetables), GF cereals and pseudo cereals, and specifically formulated substitutes of cereal-based products (e.g., bread, pasta, biscuits, cakes, and ready-to-eat meals) with a gluten content lower than 20 ppm, as stated in the European Implementing Regulation (EU) no. 828/2014 [3]. Such formulated GF products should adequately replace gluten-containing (GC) products in the diet of celiac patients, as GC cereals and their derivates represent an important energy and nutrient source in Italy [4] and worldwide [5].

Manufactured GF products’ ease of access has dramatically increased in the last decade with GF products available in mass retail channels [6]. Such products are largely consumed by celiac disease patients [7] but also to some extent by the general population [8,9]. As a result, a food composition database (FCDB) containing data on the GF cereal-based products available on the market is needed to evaluate the energy and nutrient intakes of GF foods’ consumers and the nutritional adequacy of the GF diet which is still debated. In this regard, our group was the first to develop a GF-FCDB attempting to calculate micronutrient composition. In 2015, we published the first version of the Italian FCDB of GF products [10], which reported the composition of 60 GF products present on the market in 2013. However, frequent updates based on international standards [11] are needed in FCDB to compile quality data representing the composition of foods in a specific time period and region of interest. This is particularly true for databases containing data on GF products whose formulations and nutritional values may considerably change over time due to the constant advance in food technology [12].

Previous studies have shown that GF foods and “free-from”-labelled food products are perceived as healthier than their traditional counterparts by the general population regardless of their declared nutritional composition [13,14]. The misperception of the healthiness of manufactured food products may lead to the overconsumption of such products, regardless of their nutritional quality [15] and the low consumption of naturally GF foods [16]. According to recent Italian studies, celiac patients consume higher quantities of biscuits and bread substitutes such as crackers [17] and are less adherent to the Mediterranean diet [16,18] than their healthy counterparts. Regarding macro- and micro-nutrient intake, celiac disease adults have shown higher fat and sugar intake, lower fiber intake, and lower micronutrient intakes [19,20,21]. As a result, poor vitamin D, calcium, iron, vitamin B12, and folate status has been detected in several studies in patients following a long-term GF diet [21]. However, the low adequacy of the GF diet may be the result of both the overall dietary habits of celiac patients and the inadequacy of the nutrient composition of GF food substitutes. When comparing hypothetical menus developed based on MyPlate guidelines using GC foods and the corresponding GF substitutes, the GF menu was significantly lower in protein, magnesium, potassium, vitamin E, folate, and sodium, with suggestive lower calcium and higher lipids [22]. However, a similar approach has never been applied in Italy.

The first aim of the present work was to present an updated version of the Italian GF-FCDB to represent the composition of GF products available on the market in 2020–2021 in Italy. The second aim was to compare theoretical daily energy and nutrient intakes provided by a weekly food-based dietary guideline menu replacing GC with GF cereal-based products. Since at present there is no specific dietary guideline for celiac patients in Italy, the food-based recommendations applied are those for the general population [23].

## 2. Materials and Methods

### 2.1. Update of the Gluten-Free Food Composition Database

To search for the GF products available on the Italian market in 2020–2021, we started by consulting the Nielsen database of GF products (the Nielsen, US, 2013–2014) that was integrated with the cereal-based product selection from the major retailers present in the Italian market offering a home-shopping section in their website, deeply described elsewhere [24,25,26,27]. The five brands identified in the previous version of the database [10] were integrated with new GF specialized brands (*n* = 2) and non-specialized national brand leaders (*n* = 5) that entered the market recently. When less than 2 products of the selected 12 brands were found for a food item present in the original database [10], other minor brands from the online marketplace were considered. All products considered for the update were certified with the Crossed Grain Trademark. Products that presented an incomplete or incoherent ingredient list and/or nutritional declaration within the brand or supermarket website were excluded.

The food categories considered for the update were: cookies, breakfast products, sweet products, breads, pizzas, savory snacks, flours, and pasta dishes. A new “ready-to-eat dishes” category was created in response to the presence on the online marketplace of such products. Each food category included different GF food items, representing the composition of multiple GF food products from different brands having similar characteristics.

To estimate the complete composition of the selected GF manufactured products, we used ingredients list to develop a recipe [10]. The nutrient composition of each ingredient was mainly derived from the Italian FCDB for epidemiological studies [28]. When an ingredient was not present in the latter database (e.g., leavening agents, gums, and protein isolates), the composition was estimated from calculations, or borrowed from international FCDBs [29,30]. Missing data in the composition of cereal and cereal-based ingredients were compiled in accordance with standard methodology [28].

To calculate the nutrient composition of each GF product, ingredient weights were imputed based on their ranking and percentage contribution (if available) stated on the label. The ingredients’ weights were adjusted using a trial-and-error approach until the calculation results matched the mandatory nutritional declaration (the European Regulation (EU) no. 1169/2011) [31]. The sum of all the ingredients considered the possible loss in water due to food processing. When applicable, the micronutrient composition of each ingredient was adjusted for losses due to heating or other food processing steps [32].

The described process was repeated for all branded foods collected within a single GF food item. The final nutrient composition of each food item was calculated combining the mean nutritional label data and the mean data calculated as above described. Further calculations were performed to adjust the overall nutrient composition, such as proportioning the animal and vegetable protein/lipids estimated from the recipe calculations on the total protein/lipids from the nutrition label; proportioning the amino acids, fatty acids, and single sugars on total protein, lipids, and soluble carbohydrates, respectively; recalculating starch by the difference from available and soluble carbohydrates. In addition, the energy content was calculated based on the macronutrient specific conversion factors, including fiber [28,33]. The water content was calculated by difference, or analytically determined following the official method [34] for 42 food items (over 108 total items). Finally, informatic checks were performed to control for possible errors and/or omissions.

### 2.2. Energy and Nutrient Content Evaluation of Food-Based Guidelines Menus

Based on the methodology proposed by Taetzsch and colleagues [22], we performed a theoretical analysis to estimate the daily energy and nutrient content in Italian weekly reference menus for a 2000 kcal daily diet [23] containing either GF or GC foods. Complex recipes were disassembled into simple ingredients [35]. Three scenarios were developed: The Refined Cereals (RC) scenario, the Wholegrain Cereals (WC) scenario, and the Mixed Cereals (MC) scenario. The MC menu was developed considering the recommendation [23] to prefer wholegrains and to vary the food choices (we used about 60% wholegrain and 40% refined cereal-based products). The other two menus contained only refined (RC) and wholegrain (WC) cereal-based products. The complete food list with the identified GF and GC food equivalences is reported in Table S1. The food list is identical in each scenario apart from cereal-based products. Each scenario was developed in double using traditional GC foods and the corresponding GF substitutes, respectively. The nutrient composition calculations on the GC menus were performed using the Microdiet software (Microdiet software version 4.1—Downlee Systems Ltd., High Peak, UK), which contains the Italian FCDB for epidemiological studies [28]. The newly updated GF database was then applied to replace the GC cereal-based products with the most equivalent GF substitutes in the Microsoft Excel export of GC menus.

### 2.3. Statistical Analysis

To compare the energy and nutrient content of the different GC and GF scenarios, we determined the theoretical daily intake of energy (calculated from macronutrients, including fiber) and 30 food components having no missing data in the Italian database [28]. The distribution of each nutrient per day was evaluated with the Shapiro–Wilk test for normality. A paired *t*-test and Wilcox signed-rank test were used to evaluate the differences between the GC and GF menus for normally and non-normally distributed nutrients, respectively. Statistical significance was set at *p* < 0.05.

## 3. Results

### 3.1. Update of the Gluten-Free Food Composition Database

The database includes the composition per 100 g of 108 GF foods, as sold, in terms of energy and the full range of macro- and micro-nutrients present in the latest version of the Italian FCDB [28]. The energy content and macronutrient composition are presented for: 17 GF cookies and 4 GF breakfast products (Table 1); 11 GF cakes and desserts, 21 GF sweet snack products (Table 2); 13 GF breads, 4 GF pizzas, 10 GF savory snacks, and 5 GF flours (Table 3); 16 GF pasta dishes and 7 GF ready-to eat dishes (Table 4). The fatty acids and micronutrient composition of the GF items are shown in Tables S2 and S3, respectively.

Chocolate-coated wafers had the highest content of energy, lipids, and soluble carbohydrates, and the lowest content of available carbohydrates among all GF biscuits (Table 1). The protein content was the highest in “cantucci”, while chocolate wafers had the highest saturated fatty acids (SFAs) (Table S2). Among all GF biscuits, wafers had the lowest sodium content (Table S3). Muesli had the highest soluble carbohydrates, lipids, protein, SFAs, and energy content but the lowest sodium content among all GF breakfast products (Table 1, Table S2 and S3).

In the GF cakes and desserts category (Table 2, Table S2 and S3), “tiramisù” had the highest lipid and SFAs content, and the lowest PUFAs. Sponge cake had the highest protein content and the lowest SFAs and sodium content. The highest PUFAs and sodium content were found in chocolate cake. Chocolate-coated snack bar showed the highest energy, lipid, and SFAs content among all the GF sweet snacks (Table 2 and Table S2). Sodium content was the lowest in “cornetto” ice cream, and the highest in puff pastry, plum cake, and plain muffin (>300 mg/100 g) (Table S3). Plain muffin had the highest PUFAs (Table S2).

Hamburger/hotdog-type bread had the highest soluble carbohydrates and the lowest fiber content among GF breads (Table 3). Rustic bread with seeds had the highest content of protein, lipids (Table 3), and PUFAs (Table S2). All breads contained less than 2.2 g/100 g SFAs and more than 1.4 g/100 g PUFAs. The sodium content was more than 400 mg/100 g in all breads (Table S3).

GF cooked pizza dough and “focaccia” had the highest energy and available carbohydrates and the lowest water content in the GF pizzas category (Table 3). In addition, they had lower protein than other pizzas. “Focaccia” had the lowest SFAs content (Table S2) and the highest sodium content (Table S3), while “calzone” had the highest SFAs content and the lowest sodium content.

In the GF savory snacks category (Table 3 and Table S2), crackers snacks and “taralli” had the highest lipids, and, together with wholemeal breadsticks, the highest PUFAs. Wholemeal crackers had the highest fiber and the lowest sodium content (Table S3). Saltines snacks contained the highest amount of sodium. GF flour for pasta had the highest protein and the lowest lipid content in the GF flours category (Table 3), while rustic flour had the highest lipid, fiber, and sodium content (Table S3).

GF pasta dishes (Table 4, Table S2 and S3) included gnocchi, dry pasta, fresh pasta, and filled pasta. The latter pasta type had the highest sodium and lipid content. Gnocchi had the lowest lipid and protein content. Legume pasta had the highest protein content.

Finally, there was great variability in the composition of GF ready-to-eat products (Table 4, Table S2 and S3), due to the different types and formulations of foods included.

### 3.2. Comparison between Gluten-Free and Gluten-Containing Guideline Menus

Table 5 shows the mean/median daily energy and nutrient content of the GC and GF equivalent one-week guideline menus calculated for each scenario (RC, MC, and WC).

No significant differences were found in the macronutrient composition of the MC and WC scenario, except for lipids, which were significantly higher in the GF version of the WC scenario than in the corresponding GC menu. A suggestive trend for higher lipid content in the GF alternative menu was also observed in the MC scenario. On the other hand, in the RC scenario, the GF alternative menu was significantly lower in available carbohydrates and starch content, with a suggestive trend for lower energy content than in the GC menu (*p* = 0.054 and *p* = 0.067 for energy expressed in kcal and kJ, respectively).

The main differences between GF and GC corresponding menus were observed in the fatty acid composition, particularly in the WC scenario. The GF menu of the WC scenario was higher than the corresponding GC menu in MUFAs, PUFAs, oleic, and linoleic acids, with a suggestive trend also for higher linolenic acid. Statistically significant differences were also observed between the GF and GC menus in the MC and RC scenarios regarding PUFAs and linoleic acid content.

The GF alternative menu of each scenario showed a micronutrient composition similar to the corresponding GC version, except for vitamin E, which was significantly higher in all GF menus, and zinc, which was significantly lower in the GF version of the WC scenario.

## 4. Discussion

The present paper presents a revised version of the Italian GF-FCDB and applies it to evaluate the nutritional adequacy of a theoretical GF diet. For this purpose, GC products were substituted with GF counterparts in the weekly reference menu for a 2000 kcal/day diet compiled using the recommendations of Italian food-based dietary guidelines [23]. This update was justified by the quick changes in the formulation of GF products consequent to the huge research in this field. The ongoing development of such products in turn replies to the high demand of the food market.

### 4.1. The Gluten-Free Food Composition Database

The first version of the Italian GF-FCDB originated from the need to develop a suitable database to assess the nutritional intakes of celiac disease patients in Italy. That was necessary since limited nutritional composition data of GF foods were available in FCDBs. Moreover, previous dietary assessment studies did not clearly describe the process applied to calculate the composition of GF products used to estimate nutritional intakes in celiac disease patients [10]. At present, most dietary assessment studies used GF food composition data from published GF-specific FCDBs [36,37] from databases collecting only mandatory nutrient label data [38] or from imputation procedures [39,40]. However, a large amount of missing data in the micronutrient composition of GF foods may lead to biases in the dietary assessment, especially when the missing data are in the frequently consumed foods. Recently, some national databases have included a few analytical values for a limited list of GF products, mainly bread [30,41,42,43]. Up to now, other GF label-based FCDBs have been published [12,44,45], and several studies have collected and compared GF and GC food composition data declared on nutrition labels in several countries [46,47,48,49,50]. However, only Missbach and colleagues [44] and Jamieson and colleagues [45] presented in their database a comprehensive list of nutrient values imputed from the ingredient list reported on the label using an approach comparable to ours.

The approach proposed in the present work slightly modifies the one proposed in the first version of the database. First, recipes were created using ingredients from the Italian FCDB for epidemiological studies in its new version [28], and the residual missing data on cereal-based ingredients were filled using standard methods [11]. The present GF-FCDB is still based on the ingredient list reported on the food label and it uses retention factors to calculate nutrient losses due to cooking procedures, as described elsewhere [10]. However, contrarily to the previous version, the target values from the mandatory nutrition labels were used as reported on the label and recalculated only on analytically determined dry matter, when available. Indeed, another key difference is that in the present GF-FCDB version, we performed analytical moisture determinations for 42 items (i.e., breads (*n* = 10), pizzas (*n* = 3); pasta dishes (*n* = 3), cookies (*n* = 7), cakes and desserts (*n* = 6), sweet snacks (*n* = 12), and savory snacks (*n* = 1)). Knowing the water content in food is very important because variation in water content is the main determinant of the content of other components [33]. Moreover, it is known that the hydrocolloids (mainly hydroxypropyl methylcellulose, xanthan gum, psyllium, and guar gum), which are commonly used to compensate for the lack of the gluten structure in GF products, hold a huge amount of water, leading to higher water content in GF than in GC corresponding foods [51]. In addition, water was the first ingredient on the declared list in more than 70% of the GF products used to create the bread items of the present database. As a result, the water content of traditional wheat products cannot be used to adjust the nutrient composition of the corresponding GF food items in FCDBs, and since nutritional label accuracy is mostly unknown [52], it may also be inaccurate to calculate water by difference from macronutrients.

The supply of GF products largely increased in the past decade. We found several products labeled as wholemeal, a great variety of formulations among the same food items, and several traditional products, such as “taralli”, “friselle”, “colomba”, “cantucci”, and “canestrelli”, that were not present in the previous version of the database. Overall, the macronutrient contents of GF food items, which were already present in the previous GF-FCDB, did not considerably change, with some exceptions. In biscuits, we observed a trend in the increased content of fiber, calcium, potassium, phosphorus, and folates. Moreover, there was a huge increase in the item number (more than twice). Wafers and biscuits of different types and flavors were added to the current database. This great increase in number reflects the high consumption of cookies by celiac patients reported in a previous Italian study [17]. Similarly, the sweet products category of the previous version of the database changed substantially in terms of item numerosity (32 vs. 18 products in current and previous GF-FCDB, respectively). As a result, in the current database, sweet products were divided into two categories: cakes and desserts, and sweet snacks. The added items were tarts, and different versions of same products. Sweet snacks and cakes showed a similar or higher fiber content (except for puff pastry) and lower lipids (except for “pandoro”, “panettone”, plain muffins, chocolate, and jam pastries) in the current version than in the previous GF-FCDB. Bread and savory snacks items generally had higher fiber content compared to previous similar items, except for wholemeal bread, whose fiber content decreased from 6.4 g/100 g to 5.1 g/100 g. Breads also contained more calcium and phosphorus, and in some cases more iron and zinc, than in the first version of the database. Furthermore, we observed a very variable composition among foods of the same group due to different formulations, as previously observed [53]. GF products are highly differentiated to meet consumer preferences and needs [6]. As an example, dry pasta composed of cereals such as rice, corn, and mixed cereals showed a similar macro and micronutrient composition as in the previous GF-FCDB. However, new pasta types including legumes in their formulations are now widespread on the market [24]. As an example, in the present database, legume pasta and pasta with mixed cereal and legumes were created from 12 and 4 branded product labels, respectively. The use of legumes in GF pasta formulations may substantially enhance the nutritional profile. Indeed, pasta with legume flours has a very different composition than traditional GF pasta with more proteins, fiber, minerals, and vitamins [24,54].

The main limitations of the present GF-FCDB are the prevalent use of non-analytical data, and the impossibility to apply the recipe method to all GF products available on the market. As an example, GF beers, which may be reported on a dietary record of a celiac disease patient, are not included in the present database. Moreover, the database does not include data on the non-specifically formulated GF products where cereal flours are only used as additives. However, errors in nutrient intake estimations using the composition of the corresponding GC products will plausibly be negligible due to infrequent consumption (in the case of beer) or irrelevant differences in the composition (in the case of non-specifically formulated GF products). Despite these limitations, the main features of the present database are the comprehensiveness of nutrient composition derived from standardized calculation procedures, the use of retention factors to adjust nutrient composition accounting for losses during processing, and the large sample of branded products from multiple brands considered for items construction. As an example, biscuit composition was derived from at least 4 different branded products—for coconut biscuits, “cantucci”, and “canestrelli”— up to 21 different products—for chocolate and plain biscuits. In the case of bread with olives, the composition was obtained from 2 products, whereas 15 different branded products were used for sliced white bread. Furthermore, in the literature, there is limited knowledge of the micronutrient composition of GF foods [55], and the present database is the only one representing the complete composition of GF products available in Italy. Since product availability and formulations may change over time because the GF market is fast changing, the present database provides an important update allowing its use in present-day dietary assessment studies.

### 4.2. Nutritional Adequacy of a GF Menu from General Dietary Guidelines

Previous studies showed that GF diets were generally ineffective in counteracting the mineral and vitamin deficiencies observed at diagnosis in celiac disease patients [56,57]. In addition, celiac patients adhering to a strict GF diet have lower micronutrient intakes than their healthy counterparts [19,20,21]. However, the micronutrient composition of manufactured GF foods is mostly unknown, so caution must be taken to draw conclusions on micronutrient intakes in celiac disease patients [20].

In the present theoretical assessment, when the Italian dietary guidelines were used to plan one-week GF menus, only a few differences were found between the nutrient intakes provided by the GC and GF menus. The main differences were observed in the content of lipid components, particularly in PUFAs, linoleic acid, and vitamin E.

The GF menus contained more total lipids than the corresponding GC menus in the WC scenario, with a suggestive trend for higher lipids also found in the MC scenario. Differences in lipid composition between GF and GC menus are probably due to the high recommended frequency of bread consumption, which has been previously found to contain more lipids in its GF version than its GC counterpart [25,53,58]. Indeed, fat is commonly used as an ingredient in GF leavened products to enhance the texture of the crumb, stabilize the gas bubbles in the dough, and minimize starch retrogradation [51]. In a similar theoretical assessment, Taetzsch and colleagues [22] found a suggestive trend of higher lipids in GF MyPlate menus than in the corresponding GC menus. Accordingly, most of the previous dietary assessment studies reported a higher lipid intake in celiac patients than in their healthy counterparts [20,21], except for a very recent study on the National Health and Nutrition Examination Survey (NHANES) [59]. Some previous studies on celiac disease patients also observed a higher SFAs intake compared to their healthy counterparts [20]. On the contrary, in the present theoretical assessment, the SFAs content was similar in GF and GC menus, but the PUFAs content was higher in all GF scenarios, and the MUFAs were higher in the GF-WC scenario. This may be explained by the higher MUFAs and PUFAs contents of the GF manufactured products than those of the corresponding GC foods [28]. In addition, in the present assessment, linoleic acid was found to be significantly higher in GF than in GC menus in each scenario. As a result, the PUFAs’ percentage contribution to daily energy intake was found to meet the Italian reference values (5–10 %E) [60] only in GF menus (ranging from 5.2 to 6.5 %E, in the RC and WC scenario, respectively).

Protein content was above the Italian population reference intake (PRI) [60] in all GC and GF scenarios. On the contrary, in the theoretical assessment performed by Taetzsch and colleagues, the GF diet was significantly lower in protein than the GC diet [22]. Previous studies reported an overall lower protein content of GF manufactured foods than their GC counterparts [24,25,26,49]. As a result, the adequate protein content of the GF menus found in the present assessment is likely dependent on the considerable presence of other protein sources in the guideline menu. Therefore, Italian omnivorous celiac disease patients may easily reach the recommended protein intake even if cereal-based products are known to be important protein sources [4]. More caution is needed when protein sources of animal origin are limited or excluded, as in the case of vegetarian or vegan celiac disease patients.

Independently from the cereal-type scenario, the fiber recommendation [60] was met mainly due to the high fruit and vegetable frequency of consumption indicated in the guideline menu. On the contrary, most dietary assessments on celiac patients found inadequate fiber intake and high soluble carbohydrate intakes [20,21], which may be explained, for example, by a low intake of fruit [16] and a high consumption of processed products [17].

In the present theoretical application of the GF-FCDB on the one-week dietary guidelines menu, we observed that it is possible to meet the micronutrient requirements proposed for the adult Italian population [60] by simply substituting GC cereal-based products with the corresponding GF alternatives. However, we found that in the GF diet, attention should be paid regarding zinc and iron intakes, irrespectively of the considered scenario. The zinc average requirement (AR) (10 mg/day), but not the PRI (12 mg/day) recommended for men [60], was met in the GF version of the guideline menus. Similarly, the iron AR (10 mg/day), but not the PRI (18 mg/day) recommended for premenopausal women [60], was met in the GF menus. It is also worth mentioning that the iron content was lower than the PRI for young women also in GC menus, excluding the WC scenario. On the other hand, the zinc content was found to be significantly lower in GF than in GC menus only in the WC scenario. This difference reflects the lower zinc content in wholemeal GF items than in traditional wholemeal bread; in the present GF-FCDB, the zinc content of wholemeal bread and pasta was approximately half that in the corresponding items of the Italian FCDB [28]. In addition, the imputed iron and zinc content of the GF items included in the present GF-FCDB was comparable to recent analytical data [55,61].

No differences were found between the GF and GC menus of each scenario on other critical nutrients, such as calcium and folates, which are notoriously deficient in celiac patients [21]. Therefore, the role of GF food micronutrient composition in patients’ deficiencies is likely marginal. To support this statement, micronutrient deficiencies were found in long-term GF diets mainly as a result of unhealthy dietary habits [62].

Finally, it must be noted that the sodium content was alarmingly high in each scenario, ranging from 2947 mg/day (about 7.4 g of salt per day) to 3288 mg/day (about 8.2 g of salt per day) in the GF-RC scenario and in the GC-RC scenario, respectively. This value is anyway higher than the one indicated as the upper limit of the suggested dietary target for sodium (<2000 mg/day) [60] and likely depends on the excessive content of salt found in both GC and GF cereal-based manufactured products [25,63]. Indeed, in the present GF-FCDB, no bread item met the World Health Organization (WHO) benchmark for sodium content in leavened breads, set at 330 mg/100 g [64]. Since Italian guideline menus for the general population present a considerable daily consumption of bread, product reformulations are needed to achieve WHO benchmarks and consequently reduce dietary sodium intake in the Italian population [64].

## 5. Conclusions

FCDBs for epidemiological purposes require good quality and comprehensive food composition data. The present updated database is the only one to provide a comprehensive overview of the macro- and micronutrient composition of GF manufactured products representative of GF food items available on the Italian market. The use of standardized and well-documented procedures allowed us to obtain a reliable database for epidemiological studies. Using the updated GF-FCDB to perform a theoretical assessment of the dietary intakes of a GF diet based on Italian guideline menus for the general population, we observed that it is possible for celiac disease patients to meet nutrient requirements by simply substituting GC foods with GF cereal-based products. Therefore, more effort should be put into educating celiac disease patients in following the dietary recommendations. This updated GF-FCDB will be a useful tool for assessing the nutrient adequacy of the actual diet of the Italian celiac disease population and for spotting potential nutrient deficiencies.

## Figures and Tables

**Table 1:** Energy and macronutrient composition per 100 g of GF biscuits and breakfast products, as sold.

	Energy (kJ)	Energy (kcal)	Water (g)	Av. Carb. (g)	Sol. Carb. (g)	Fiber (g)	Protein (g)	Lipids (g)
BISCUITS								
Biscuits, “canestrelli”	1850	440	7.9	67.7	22.5	1.4	3.8	18.7
Biscuits, “cantucci”	1899	452	2.4	64.1	26.0	4.3	9.9	18.1
Biscuits, “cantucci”, with chocolate	1666	395	9.1	69.9	33.0	3.4	5.1	11.8
Biscuits, chocolate-coated	2034	485	2.2	63.4	35.7	3.6	5.5	24.2
Biscuits, ladyfinger	1622	383	4.8	77.3	35.7	2.9	7.4	6.4
Biscuits, plain	1882	447	2.3	73.5	22.0	1.9	4.4	16.7
Biscuits, wholemeal	1959	467	2.0	67.2	20.2	5.5	6.0	18.1
Biscuits, with chocolate	1873	446	7.5	66.6	24.8	3.2	4.7	17.2
Biscuits, with coconut	1921	457	4.7	68.2	21.0	2.1	4.3	20.0
Biscuits, with jam	1636	388	13.7	66.2	27.2	2.8	3.0	13.6
Breakfast biscuits	1855	440	0.9	75.9	19.2	2.8	4.2	14.8
Filled biscuits	1995	476	3.4	66.0	34.4	2.5	4.2	22.9
Tea biscuits	1977	471	2.3	68.5	27.4	2.5	4.5	21.2
Wafers, chocolate	2136	511	1.8	60.2	26.8	3.9	4.3	28.9
Wafers, chocolate-coated	2309	554	1.5	55.6	39.2	2.7	5.4	33.8
Wafers, hazelnut	2110	504	1.9	62.8	24.3	2.8	4.0	27.4
Wafers, vanilla	2073	494	0.7	69.2	30.3	1.8	3.2	24.3
BREAKFAST PRODUCTS								
Cereal rusks	1556	368	6.0	74.3	2.4	6.4	9.2	2.3
Melba toast	1608	380	4.0	75.2	5.7	6.6	4.4	7.5
Melba toast, wholemeal	1699	403	4.0	74.3	4.2	7.9	3.9	8.3
Muesli	1657	394	12.1	57.7	20.2	5.3	9.5	14.3

Abbreviations: Av. Carb, available carbohydrates; Sol. Carb, soluble carbohydrates.

**Table 2:** Energy and macronutrient composition per 100 g of GF cakes, desserts, and sweet snacks, as sold.

	Energy (kJ)	Energy (kcal)	Water (g)	Av. Carb. (g)	Sol. Carb. (g)	Fiber (g)	Protein (g)	Lipids (g)
CAKES AND DESSERTS								
Cake, “colomba”	1802	430	15.0	53.7	30.9	2.8	4.9	22.7
Cake, “margherita”	1519	362	22.9	53.3	27.0	3.8	3.5	15.6
Cake, “pandoro”	1534	366	26.5	46.7	18.7	3.1	4.3	18.6
Cake, “panettone”	1390	331	29.6	47.1	24.5	4.0	4.1	14.4
Cake, “panettone”, with chocolate	1382	329	29.6	47.2	19.9	3.7	5.0	13.8
Cake, chocolate	1729	413	19.0	49.5	26.5	3.5	4.5	22.5
Dessert, “tiramisù”	1256	302	49.9	19.5	16.0	2.8	4.2	23.0
Sponge cake	1203	285	34.3	49.4	28.2	1.3	6.6	7.8
Sweet bread	1141	271	37.0	49.0	12.6	4.2	2.9	6.0
Tart, with chocolate and hazelnut	1469	350	28.2	47.1	25.8	2.5	4.9	16.6
Tart, with jam	1623	385	12.5	69.9	32.3	1.8	3.4	11.7
SWEET SNACKS								
Croissant	1366	325	27.9	50.6	16.4	4.5	3.7	12.4
Croissant, with chocolate	1337	318	31.1	47.7	14.8	4.0	2.9	13.3
Croissant, with jam	1262	300	33.4	48.1	17.6	3.7	2.8	11.2
Ice cream, “cornetto”	1293	309	39.8	36.3	23.3	2.8	3.6	17.0
Ice cream, sandwich-type	1277	304	36.3	45.0	23.8	1.0	4.0	13.0
Muffin, plain	1669	398	19.1	52.6	26.3	2.8	4.5	19.7
Muffin, with chocolate	1999	477	5.1	63.5	30.5	1.4	4.9	24.0
Muffin, with fruit	1670	398	17.5	55.9	29.3	3.3	4.0	18.4
Pastries, plain	1807	432	16.6	52.7	27.7	2.4	5.7	21.5
Pastries, with chocolate	1737	414	17.1	52.6	31.3	2.8	5.4	21.1
Pastries, with jam	1564	372	19.9	58.5	32.3	2.3	3.9	14.7
Pastries, with milk	1594	379	20.3	57.0	33.8	1.0	4.4	16.2
Pastries, without added sugars	1627	388	19.8	50.8	1.1	4.8	4.8	18.8
Plum cake	1727	412	17.9	52.6	23.8	2.2	4.9	21.2
Plum cake, with chocolate	1609	384	25.8	44.4	23.7	2.6	4.6	21.6
Puff pastry	1500	360	34.6	33.5	1.6	4.4	2.4	24.0
Snack bar, cereals and chocolate	1661	395	9.2	60.6	29.4	9.8	5.6	14.0
Snack bar, cereals and nuts	1725	409	6.3	69.0	37.7	3.2	8.0	12.4
Snack bar, chocolate-coated	2146	513	2.2	57.7	40.3	3.4	6.5	29.3
Snack roll, “cannolo”	1944	463	5.3	66.7	34.6	2.9	5.7	18.6
Wafer cone, for ice cream	1690	400	1.2	80.1	17.0	7.2	5.6	4.8

Abbreviations: Av. Carb, available carbohydrates; Sol. Carb, soluble carbohydrates.

**Table 3:** Energy and macronutrient composition per 100 g of GF breads, pizzas, savory snacks, and flours, as sold.

	Energy (kJ)	Energy (kcal)	Water (g)	Av. Carb. (g)	Sol. Carb. (g)	Fiber (g)	Protein (g)	Lipids (g)
BREADS								
“Piadina”	1268	301	29.5	53.8	2.5	4.3	3.1	7.2
“Piadina”, wholemeal	1234	293	29.5	51.2	0.5	6.8	3.3	6.8
Bread, “ciabatta”, “baguette”, “sfilatino”	1169	277	33.5	49.8	4.5	5.8	3.7	5.7
Bread, “rosetta”, “tartaruga”	1062	252	36.8	48.9	3.0	6.3	3.1	3.5
Bread, hamburger/hotdog type	1047	248	39.6	46.4	6.5	3.0	3.0	4.6
Bread, prepared with oil	1236	294	33.0	48.7	3.0	5.0	3.6	8.3
Bread, rustic, with seeds	1136	271	37.2	38.5	4.1	8.5	5.6	8.6
Bread, white, sandwich-type	1057	251	40.1	43.8	5.1	5.5	3.3	5.7
Bread, white, sliced	1112	264	37.2	45.4	4.0	5.9	3.9	6.2
Bread, wholemeal	1151	274	37.2	44.1	4.6	5.1	4.1	7.8
Bread, with olives	1103	263	38.5	40.6	4.3	7.3	4.2	7.7
Breadcrumb	1578	374	10.0	71.2	2.7	5.5	5.5	6.2
Tortilla wrap	1191	283	29.5	47.8	1.9	10.3	3.4	6.4
PIZZAS								
“Calzone”, frozen	871	207	54.1	27.5	2.1	2.6	6.3	8.2
“Focaccia”	1243	296	30.9	49.4	4.9	5.9	2.9	8.3
Pizza dough, cooked	1324	314	27.3	55.1	1.6	4.2	3.0	8.2
Pizza, tomato and mozzarella	943	224	49.2	31.2	2.5	2.9	6.8	8.3
SAVORY SNACKS								
“Friselle”	1580	374	4.9	77.7	10.7	7.2	4.0	3.6
“Taralli”	1984	474	3.8	64.0	2.1	3.9	3.3	21.9
Breadsticks	1761	418	3.8	77.2	3.0	3.2	3.4	9.9
Breadsticks, wholemeal	1874	446	2.7	72.7	3.7	4.1	2.7	15.1
Cheese and cereals snacks	1651	391	4.4	73.4	2.3	5.8	8.3	5.8
Crackers snacks	2023	482	3.3	71.5	2.5	2.8	2.5	20.1
Crackers, salted	1867	444	2.1	75.0	3.1	3.1	4.1	13.5
Crackers, wholemeal	1784	425	3.6	65.7	1.9	9.5	5.7	13.3
Croutons	1651	392	10.2	69.9	3.5	5.0	2.6	10.2
Saltines snacks	1801	428	2.0	74.5	2.7	3.6	3.3	12.1
FLOURS								
Flour, for bread and pizza	1401	329	10.4	81.8	3.7	3.3	2.6	0.6
Flour, for cakes	1430	336	10.2	81.9	11.4	3.1	3.2	1.1
Flour, for pasta	1425	335	9.6	81.8	2.8	3.0	4.3	0.5
Flour, rustic	1341	316	13.0	70.5	3.3	8.8	2.6	1.8
Flour, unspecified	1405	330	10.5	81.8	3.4	4.1	2.4	0.7

Abbreviations: Av. Carb, available carbohydrates; Sol. Carb, soluble carbohydrates.

**Table 4:** Energy and macronutrient composition per 100 g of GF pasta and ready-to-eat dishes, as sold.

	Energy (kJ)	Energy (kcal)	Water (g)	Av. Carb. (g)	Sol. Carb. (g)	Fiber (g)	Protein (g)	Lipids (g)
PASTA DISHES								
“Ravioli”, filled with meat	1397	333	31.2	38.2	0.3	1.8	12.2	15.3
“Ravioli”, mixed fillings, fresh	694	165	63.5	23.6	1.0	1.3	4.4	6.3
“Ravioli”, spinach and ricotta cheese	1116	265	37.7	40.4	3.5	2.9	8.9	8.0
“Tortellini”, filled with meat	1244	295	31.2	43.2	1.0	3.0	10.2	9.6
Cous cous	1464	345	9.0	72.3	1.0	5.1	10.9	2.2
Egg pasta, dry	1498	353	10.5	74.4	0.3	2.1	8.0	4.2
Egg pasta, fresh	1052	249	36.9	45.2	0.1	5.5	7.1	4.4
Gnocchi	658	155	56.8	36.0	0.5	2.0	3.1	0.3
Legume pasta	1383	327	12.7	51.3	2.0	9.2	22.4	2.9
Pasta, buckwheat	1402	330	14.2	67.0	0.9	4.5	11.0	2.9
Pasta, corn	1449	340	10.0	79.4	0.6	2.2	6.9	1.2
Pasta, for broth	1426	335	11.8	76.8	0.6	2.5	6.6	1.8
Pasta, mixed cereals	1422	334	12.5	75.4	0.7	2.6	7.2	1.9
Pasta, mixed cereals and legumes	1427	336	11.9	70.4	0.9	4.3	9.5	2.8
Pasta, rice	1442	339	11.5	77.6	0.7	1.4	7.5	1.7
Pasta, wholemeal	1433	337	11.8	74.4	0.7	3.4	7.3	2.5
READY-TO-EAT DISHES								
“Lasagne” with meat	600	143	70.1	15.9	2.3	0.5	6.0	6.5
Breaded cheese, frozen	722	171	59.6	24.5	4.8	2.0	6.4	5.6
Chicken breast, breaded, frozen	966	231	54.4	18.0	0.5	0.5	13.0	12.2
Fish, breaded, frozen	840	201	60.2	15.6	1.1	1.2	10.7	10.8
Pasta with pesto sauce	759	180	57.1	31.6	0.6	2.9	2.9	4.9
Pasta with tomato sauce	1240	292	22.0	64.5	3.3	4.4	6.3	1.8
Soup powder, with cereals, mixed	1444	341	10.1	61.7	2.8	8.2	15.0	3.7

Abbreviations: Av. Carb, available carbohydrates; Sol. Carb, soluble carbohydrates.

**Table 5:** Daily energy and nutrient composition of gluten-free and gluten-containing one-week guideline menus for each scenario: refined cereals, mixed cereals, and wholegrain cereals.

	Refined Cereals Scenario			Mixed Cereals Scenario			Wholegrain Cereals Scenario		
	Mean ± SD/ Median (IQR)		*p* Value	Mean ± SD/ Median (IQR)		*p* Value	Mean ± SD/ Median (IQR)		*p* Value
	GC	GF		GC	GF		GC	GF	
Energy (kJ)	9839 ± 325	9417 ± 408	0.067	9602 ± 442	9405 ± 289	0.345	9496 ± 278	9431 ± 342	0.785
Energy (kcal)	2334 ± 79	2238 ± 98	0.054	2279 ± 105	2237 ± 69	0.385	2255 ± 68	2243 ± 82	0.704
Av. Carb. (g)	345.0 ± 28.1	309.1 ± 26.8	**0.031**	327.0 ± 38.8	299.9 ± 31.0	0.174	314.6 ± 28.2	299.7 ± 26.8	0.329
Sol. Carb. (g)	102.6 ± 16.7	97.7 ± 10.3	0.522	140.7 ± 19.8	98.6 ± 9.9	0.482	103.7 ± 16.7	99.5 ± 9.9	0.577
Starch (g)	*242.3* (*20.7*)	*203.3* (*5.6*)	***0.018***	222.3 ± 41.7	201.3 ± 32.7	0.313	211.0 ± 23.4	200.2 ± 27.2	0.443
Fiber (g)	37.6 ± 7.1	39.7 ± 8.5	0.625	43.1 ± 6.0	41.2 ± 8.9	0.648	48.7 ± 9.0	39.9 ± 9.0	0.089
Protein (g)	90.2 ± 14.1	79.1 ± 11.5	0.132	90.2 ± 14.5	80.8 ± 12.8	0.219	91.2 ± 14.8	80.1 ± 11.9	0.148
Lipids (g)	67.1 ± 12.3	75.9 ± 10.2	0.170	67.3 ± 9.6	78.5 ± 10.6	0.061	68.1 ± 9.5	79.9 ± 9.4	**0.039**
SFAs (g)	18.6 ± 5.8	20.9 ± 5.8	0.457	18.7 ± 5.4	21.0 ± 5.8	0.442	18.9 ± 5.3	21.2 ± 5.4	0.434
MUFAs (g)	34.0 ± 5.4	37.3 ± 4.3	0.234	33.1 ± 4.0	37.4 ± 3.9	0.064	32.7 ± 3.2	37.7 ± 3.1	**0.012**
PUFAs (g)	9.1 ± 2.6	12.4 ± 2.5	**0.031**	9.9 ± 3.1	14.6 ± 4.4	**0.040**	10.8 ± 2.9	15.6 ± 3.2	**0.012**
Oleic acid (g)	32.90 ± 5.15	36.09 ± 3.96	0.218	32.02 ± 3.81	36.25 ± 3.66	0.056	31.63 ± 3.00	36.50 ± 2.82	**0.009**
Linoleic acid (g)	7.44 ± 2.23	10.59 ± 2.19	**0.020**	8.18 ± 2.74	12.44 ± 3.94	**0.037**	9.09 ± 2.53	13.51 ± 2.85	**0.010**
Linolenic acid (g)	1.34 ± 0.35	1.50 ± 0.37	0.406	1.37 ± 0.33	1.81 ± 0.45	0.059	1.41 ± 0.36	1.80 ± 0.36	0.062
Fe (mg)	15.0 ± 4.0	14.7 ± 2.8	0.882	17.0 ± 4.1	15.7 ± 3.1	0.543	19.0 ± 4.8	15.7 ± 3.0	0.144
Ca (mg)	1311 ± 209	1266 ± 146	0.649	1281 ± 172	1282 ± 142	0.985	1268 ± 179	1274 ± 148	0.952
Na (mg)	3288 ± 845	2947 ± 752	0.441	3235 ± 874	2991 ± 749	0.585	3218 ± 818	3079 ± 775	0.750
K (mg)	4342 ± 596	4211 ± 680	0.708	4476 ± 580	4320 ± 717	0.663	4613 ± 672	4289 ± 722	0.402
P (mg)	1538 ± 260	1458 ± 160	0.505	1685 ± 345	1555 ± 205	0.409	1829 ± 317	1634 ± 199	0.193
Zn (mg)	12.19 ± 1.79	10.65 ± 1.58	0.114	13.93 ± 3.51	11.49 ± 1.90	0.132	14.55 ± 2.36	11.62 ± 1.86	**0.024**
Vitamin D (µg)	*1.01* (*0.83*)	*1.02* (*0.84*)	*0.798*	*1.01* (*0.83*)	*1.03* (*0.84*)	*0.798*	*1.01* (*0.83*)	*1.03* (*0.84*)	*0.798*
Vitamin E (mg)	14.11 ± 2.10	17.18 ± 2.02	**0.016**	14.67 ± 2.50	18.59 ± 2.42	**0.012**	14.55 ± 2.29	19.59 ± 1.82	**0.001**
Ret. Eq (µg)	*1334* (*572*)	*1417* (*640*)	*0.406*	*1334* (*572*)	*1371* (*666*)	*0.406*	*1334* (*572*)	*1348* (*652*)	*0.482*
Vitamin B1 (mg)	1.63 ± 0.54	1.56 ± 0.54	0.819	1.93 ± 0.58	1.70 ± 0.57	0.479	2.28 ± 0.50	1.86 ± 0.58	0.176
Vitamin B2 (mg)	2.17 ± 0.25	2.06 ± 0.23	0.417	2.22 ± 0.23	2.07 ± 0.21	0.251	2.28 ± 0.23	2.06 ± 0.21	0.101
Niacin (mg)	*18.46* (*15.21*)	*16.92* (*14.91*)	*0.565*	24.90 (13.53)	18.88 (13.99)	0.482	31.58 ± 13.67	27.16 ± 12.89	0.544
Vitamin B6 (mg)	2.50 ± 0.51	2.61 ± 0.54	0.700	2.62 ± 0.58	2.74 ± 0.53	0.685	2.75 ± 0.59	2.85 ± 0.54	0.732
Folates (µg)	561 ± 160	532 ± 165	0.750	569 ± 159	551 ± 142	0.828	582 ± 160	550 ± 159	0.721
Vitamin C (mg)	*211* (*141*)	*211* (*143*)	*0.749*	*211* (*141*)	*211* (*142*)	*0.749*	*211* (*141*)	*211* (*143*)	*0.749*

Abbreviations: SD, standard deviation; IQR, interquartile range; GC, gluten-containing; GF, gluten-free; Av. Carb, available carbohydrates; Sol. Carb, soluble carbohydrates; SFAs, saturated fatty acids; MUFAs, monounsaturated fatty acids; PUFAs, polyunsaturated fatty acids; Ret. Eq., retinol equivalents. A paired *t*-test and Wilcox signed-rank test were applied to detect any statistical differences between GC and GF menus for normally and non-normally distributed nutrients, respectively. Non-normally distributed nutrients are reported as median (IQR) and highlighted in italics. Significant differences are highlighted in bold typeface.

## Data Availability

The data presented in this study are available within the article and supplementary material.

## Supplementary Materials

The following supporting information can be downloaded at: https://www.mdpi.com/article/10.3390/nu14194171/s1, Table S1: Gluten-free and gluten-containing menus with substitutions for each cereal based products scenario; Table S2: Fatty acid composition per 100 g of gluten-free foods from the categories: cookies, breakfast products, cakes and desserts, sweet snacks, breads, pizzas, savory snacks, flours, pasta dishes, ready-to-eat dishes; Table S3: Micronutrient composition per 100 g of gluten-free foods from the following categories: cookies, breakfast products, cakes and desserts, sweet snacks, breads, pizzas, savory snacks, flours, pasta dishes, ready-to-eat dishes.
