# Supplementary material for: The Update of the Italian Food Composition Database of Gluten-Free Products and Its Application in Food-Based Dietary Guidelines Menus
Source: Nutrients. 2022 Oct 7;14(19):4171. doi: 10.3390/nu14194171 (PMC9571138; doi:10.3390/nu14194171)
Supplement: Supplementary file 1 [file nutrients-14-04171-s001.zip › nutrients-1910597-supplementary.pdf]

The Update of the Italian Food Composition Database of Gluten-Free Products and its Application in Food-Based Dietary Guidelines Menus

Federica Fiori [1], Maria Parpinel [1], Federico Morreale [2] and Nicoletta Pellegrini [3,*]

SUPPLEMENTARY MATERIALS

Table S1. Gluten-free and gluten containing menus with substitutions for each cereal based products scenario.

Meal	Portion size (g)	GLUTEN CONTAINING Food	GLUTEN FREE Food
DAY 1			
Breakfast	250	Semi-skimmed milk	Semi-skimmed milk
	30	*RC, MC, WC: Breakfast cookies*	*RC, MC, WC: Breakfast cookies, GF*
Mid-morning snack	225	Fresh orange juice	Fresh orange juice
Lunch	80	*RC: Pasta*<br>*MC: Pasta, wholemeal*<br>*WC: Pasta, wholemeal*	*RC: Pasta, corn, GF*<br>*MC: Pasta, buckwheat, GF*<br>*WC: Pasta, wholemeal, GF*
	50	Tomato sauce	Tomato sauce
	100	Chicken breast	Chicken breast
	63	Semi-skimmed milk	Semi-skimmed milk
	200	Zucchini	Zucchini
	75	*RC: White bread, type 00, small loaf*<br>*MC, WC: : Bread, wholemeal*	*RC: Bread, "rosetta", "tartaruga, GF*<br>*MC, WC: Bread, wholemeal, GF*
	150	Fresh fruit	Fresh fruit
Afternoon snack	15	Nuts	Nuts
	25	*RC: White bread, type 00, small loaf*<br>*MC, WC: Bread, wholemeal*	*RC: Bread, "rosetta", "tartaruga, GF*<br>*MC, WC: Wholemeal bread, GF*
Dinner	40	Rice	Rice
	350	Vegetable soup (mixed vegetables and broth)	Vegetable soup (mixed vegetables and broth)
	50	Parmesan cheese	Parmesan cheese
	80	Salad	Salad
	75	*RC: White bread, type 00, small loaf*<br>*MC, WC: Bread, wholemeal*	*RC: Bread, "rosetta", "tartaruga, GF*<br>*MC, WC: Wholemeal bread, GF*
DAY 2			
Breakfast	250	Yogurt, plain	Yogurt, plain
	150	Fresh fruit	Fresh fruit
	25	*RC: Bread, prepared with oil*<br>*MC: White bread, plain*<br>*WC: Bread, wholemeal*	*RC: Bread, prepared with oil, GF*<br>*MC: Bread, white, sliced, GF*<br>*WC: Bread, wholemeal, GF*
Mid-morning snack	225	Fresh orange juice	Fresh orange juice
Lunch	40	*RC, MC: Pasta*<br>*WC: Pasta, wholemeal*	*RC, MC: Pasta, for broth, GF*<br>*WC: Pasta, wholemeal, GF*
	150	Beans	Beans
	300	Vegetable broth	Vegetable broth

	100	Mozzarella cheese	Mozzarella cheese
	80	Salad	Salad
	150	Fresh fruit	Fresh fruit
	75	*RC: Bread, prepared with oil*<br>*MC: White bread, plain*<br>*WC: Bread, wholemeal*	*RC: Bread, prepared with oil, GF*<br>*MC: Bread, white, sliced, GF*<br>*WC: Bread, wholemeal, GF*
Afternoon snack	125	Yogurt, skimmed milk, with fruit	Yogurt, skimmed milk, with fruit
Dinner	80	*RC, MC, WC: Wheat, semolina (cous cous)*	*RC, MC, WC: Cous cous, GF*
	200	Sweet peppers	Sweet peppers
	50	Cured ham	Cured ham
	200	Fennel	Fennel
	75	*RC: Bread, prepared with oil*<br>*MC: White bread, plain*<br>*WC: Bread, wholemeal*	*RC: Bread, prepared with oil, GF*<br>*MC: Bread, white, sliced, GF*<br>*WC: Bread, wholemeal, GF*
DAY 3			
Breakfast	250	Semi-skimmed milk	Semi-skimmed milk
	30	Cornflakes	Cornflakes
	150	Fresh fruit	Fresh fruit
Mid-morning snack	25	*RC: White bread, plain*<br>*MC: Bread, wheat and rye*<br>*WC: Bread, wholemeal*	*RC: Bread, white, sliced, GF*<br>*MC: Bread, rustic, with seeds, GF*<br>*WC: Bread, wholemeal, GF*
	15	Nuts	Nuts
Lunch	80	*RC: Pasta*<br>*MC: Pasta, wholemeal*<br>*WC: Pasta, wholemeal*	*RC: Pasta, rice, GF*<br>*MC: Pasta, wholemeal, GF*<br>*WC: Pasta, wholemeal, GF*
	60	Shrimps	Shrimps
	60	Squids	Squids
	30	Clams	Clams
	200	Carrots	Carrots
	150	Fresh fruit	Fresh fruit
Afternoon snack	75	*RC: White bread, plain*<br>*MC: Bread, wheat and rye*<br>*WC: Bread, wholemeal*	*RC: Bread, white, sliced, GF*<br>*MC: Bread, rustic, with seeds, GF*<br>*WC: Bread, wholemeal, GF*
	125	Yogurt, skimmed milk, with fruit	Yogurt, skimmed milk, with fruit
Dinner	40	*RC, MC, WC: Durum wheat, grains*	*Rice*
	350	Vegetable soup (mixed vegetables and broth)	Vegetable soup (mixed vegetables and broth)
	100	Beef, lean meat	Beef, lean meat
	80	Salad	Salad
	150	Fresh fruit	Fresh fruit
	75	*RC: White bread, plain*<br>*MC: Bread, wheat and rye*<br>*WC: Bread, wholemeal*	*RC: Bread, white, sliced, GF*<br>*MC: Bread, rustic, with seeds, GF*<br>*WC: Bread, wholemeal, GF*
DAY 4			
Breakfast	125	Yogurt, plain	Yogurt, plain
	150	Fresh fruit	Fresh fruit
Mid-morning snack	50	*RC, MC: White bread, type 00, small loaf*<br>*WC: Bread, wholemeal*	*RC, MC: Bread, "ciabatta", "baguette", "sfilatino", GF*<br>*WC: Bread, wholemeal, GF*
	10	Jam	Jam

Lunch	200	*RC, MC, WC: Gnocchi (Potatoes and wheat flour)*	*RC, MC, WC: Gnocchi SG*
	50	Tomato sauce	Tomato sauce
	50	Egg, whole	Egg, whole
	200	Spinach	Spinach
	75	*RC: White bread, type 00, small loaf*<br>*MC: White bread, type 00, small loaf*<br>*WC: Bread, wholemeal*	*RC: Bread, "ciabatta", "baguette", "sfilatino", GF*<br>*MC: Bread, "rosetta", "tartaruga", GF*<br>*WC: Bread, wholemeal, GF*
Afternoon snack	125	Semi-skimmed milk	Semi-skimmed milk
	150	Fresh fruit	Fresh fruit
Dinner	120	*RC, MC: Pasta*<br>*WC: Pasta, wholemeal*	*RC, MC: Pasta, mixed cereals, GF*<br>*WC: Pasta, wholemeal, GF*
	25	Tuna, canned in olive oil	Tuna, canned in olive oil
	150	Lentils, canned	Lentils, canned
	200	Artichokes	Artichokes
	75	*RC: White bread, type 00, small loaf*<br>*MC: White bread, type 00, small loaf*<br>*WC: Bread, wholemeal*	*RC: Bread, "ciabatta", "baguette", "sfilatino", GF*<br>*MC: Bread, "rosetta", "tartaruga", GF*<br>*WC: Bread, wholemeal, GF*
	150	Fresh fruit	Fresh fruit
DAY 5			
Breakfast	250	Semi-skimmed milk	Semi-skimmed milk
	30	*RC, MC, WC: Muesli*	*RC, MC, WC: Muesli, GF*
Mid-morning snack	30	*RC: Crackers, with salt*<br>*MC, WC: Crackers, wholemeal*	*RC: Crackers, with salt, GF*<br>*MC, WC: Crackers, wholemeal, GF*
	150	Fresh fruit	Fresh fruit
Lunch	80	*RC: Pasta*<br>*MC, WC: Pasta, wholemeal*	*RC: Pasta, corn, GF*<br>*MC, WC: Wholemeal pasta, GF*
	200	Broccoli	Broccoli
	150	Cod	Cod
	50	Tomato sauce	Tomato sauce
	200	Brussel Sprouts	Brussel Sprouts
	75	*RC: Bread, prepared with oil*<br>*MC, WC: Bread, wholemeal*	*RC: Bread, prepared with oil, GF*<br>*MC, WC: Bread, wholemeal, GF*
	150	Fresh fruit	Fresh fruit
Afternoon snack	125	Yogurt, plain	Yogurt, plain
	15	Nuts	Nuts
Dinner	50	Egg, whole	Egg, whole
	40	*RC, MC: Pasta*<br>*WC: Pasta, wholemeal*	*RC, MC: Pasta, corn, GF*<br>*WC: Pasta, wholemeal, GF*
	80	Salad	Salad
	150	Fresh fruit	Fresh fruit
	75	*RC: Bread, prepared with oil*<br>*MC, WC: Bread, wholemeal*	*RC: Bread, prepared with oil, GF*<br>*MC, WC: Bread, wholemeal, GF*
DAY 6			
Breakfast	125	Yogurt, plain	Yogurt, plain
	25	*RC: White bread, plain*<br>*MC: Bread, prepared with oil*<br>*WC: Bread, wholemeal*	*RC: Bread, white, sliced, GF*<br>*MC: Bread, prepared with oil, GF*<br>*WC: Bread, wholemeal, GF*

	10	Jam	Jam
Mid-morning snack	150	Fresh fruit	Fresh fruit
Lunch	80	*RC, MC, WC: Barley*	*RC, MC, WC: Rice*
	100	Mushrooms	Mushrooms
	50	Egg, whole	Egg, whole
	200	Swiss chard	Swiss chard
	75	*RC: White bread, plain*<br>*MC: Bread, prepared with oil*<br>*WC: Bread, wholemeal*	*RC: Bread, white, sliced, GF*<br>*MC: Bread, prepared with oil, GF*<br>*WC: Bread, wholemeal, GF*
	150	Fresh fruit	Fresh fruit
Afternoon snack	125	Semi-skimmed milk	Semi-skimmed milk
	225	Fresh fruit	Fresh fruit
Dinner	300	*RC, MC, WC: Pizza, tomato and mozzarella*	*RC, MC, WC: Pizza, tomato and mozzarella, GF*
	80	Salad	Salad
DAY 7			
Breakfast	250	Semi-skimmed milk	Semi-skimmed milk
	50	*RC, MC, WC: Croissant*	*RC, MC, WC: Croissant, GF*
	150	Fresh fruit	Fresh fruit
Mid-morning snack	15	Nuts	Nuts
	25	*RC: White bread, type 00, small loaf*<br>*MC, WC: Bread, wholemeal*	*RC: Bread, "rosetta", "tartaruga", GF*<br>*MC, WC: Wholemeal bread, GF*
Lunch	100	Turkey, breast	Turkey, breast
	200	Potatoes	Potatoes
	200	Aubergine	Aubergine
	75	*RC: White bread, type 00, small loaf*<br>*MC, WC: Bread, wholemeal*	*RC: Bread, "rosetta", "tartaruga", GF*<br>*MC, WC: Wholemeal bread, GF*
Afternoon snack	125	Yogurt, plain	Yogurt, plain
	150	Fresh fruit	Fresh fruit
Dinner	80	*RC: Pasta*<br>*MC: Pasta*<br>*WC: Pasta, wholemeal*	*RC: Pasta, rice, GF*<br>*MC: Pasta, mixed cereals and legumes, GF*<br>*WC: Pasta, wholemeal, GF*
	150	Chickpeas, canned	Chickpeas, canned
	300	Vegetable broth	Vegetable broth
	80	Salad	Salad
	150	Fresh fruit	Fresh fruit
	75	*RC: White bread, type 00, small loaf*<br>*MC, WC: Bread, wholemeal*	*RC: Bread, "rosetta", "tartaruga", GF*<br>*MC, WC: Wholemeal bread, GF*

Abbreviations: GF, gluten free; RC, refined cereals; MC, mixed cereals; WC, wholegrain cereals. The menu included 30 g of extra virgin olive oil, 10 g of parmesan cheese, and 7.5 g of sugar per day. Cereal-based products are highlighted in italics.

Table S2. Fatty acid composition per 100 g of gluten free foods from the categories: cookies, breakfast products, cakes and desserts, sweet snacks, breads, pizzas, savory snacks, flours, pasta dishes, ready-to-eat dishes.

Foods	SFAs	MUFAs	PUFAs	Oleic Acid	Linoleic Acid	Linolenic Acid
	g	g	g	g	g	g
BISCUITS						
Biscuits, "canestrelli"	7.7	5.8	4.8	5.37	4.58	0.17
Biscuits, "cantucci"	5.0	9.9	4.0	9.61	3.80	0.12
Biscuits, "cantucci", with chocolate	3.5	4.1	3.0	3.92	2.89	0.04
Biscuits, chocolate-coated	14.8	7.8	1.7	7.54	1.38	0.29
Biscuits, ladyfinger	1.7	1.9	1.0	1.72	0.83	0.04
Biscuits, plain	6.4	5.6	3.5	5.36	3.11	0.33
Biscuits, wholemeal	5.5	5.8	5.4	5.56	5.14	0.23
Biscuits, with chocolate	7.1	5.6	3.5	5.40	3.28	0.21
Biscuits, with coconut	12.1	5.3	2.7	4.81	2.50	0.23
Biscuits, with jam	5.6	4.6	2.6	4.42	2.37	0.22
Breakfast biscuits	5.0	4.9	4.3	4.74	4.05	0.24
Filled biscuits	11.4	7.7	3.7	7.57	3.28	0.35
Tea biscuits	12.1	7.2	2.1	6.85	1.68	0.40
Wafers, chocolate	21.8	6.7	2.3	6.64	1.76	0.45
Wafers, chocolate-coated	19.6	12.0	2.3	11.73	1.81	0.38
Wafers, hazelnut	16.5	5.6	1.4	5.55	1.19	0.16
Wafers, vanilla	18.3	1.6	0.6	1.59	0.53	0.02
BREAKFAST PRODUCTS						
Cereal rusks	0.6	0.8	0.7	0.80	0.57	0.10
Melba toast	1.7	2.5	3.0	2.44	2.92	0.07
Melba toast, wholemeal	1.4	2.8	3.8	2.75	3.43	0.38
Muesli	4.3	5.8	3.3	5.61	2.71	0.48
CAKES AND DESSERTS						
Cake, "colomba"	4.7	7.5	9.0	7.32	8.81	0.07
Cake, "margherita"	3.9	6.0	5.0	5.71	4.87	0.10
Cake, "pandoro"	11.9	5.5	1.0	4.85	0.69	0.23
Cake, "panettone"	8.8	4.1	0.9	3.63	0.70	0.18
Cake, "panettone", with chocolate	8.4	4.1	0.9	3.69	0.71	0.15
Cake, chocolate	4.0	7.4	9.7	7.29	9.53	0.08
Dessert, "tiramisù"	16.0	7.1	0.8	6.03	0.48	0.28
Sponge cake	2.6	2.3	1.2	2.11	1.01	0.04
Sweet bread	3.4	1.7	1.1	1.66	1.03	0.10
Tart, with chocolate and hazelnut	9.2	5.2	1.1	4.66	0.88	0.18
Tart, with jam	3.2	3.7	4.6	3.57	4.55	0.06
SWEET SNACKS						
Croissant	5.5	3.5	2.7	3.43	2.44	0.19
Croissant, with chocolate	5.8	4.3	2.1	4.13	1.93	0.16
Croissant, with jam	5.1	3.1	1.9	3.03	1.73	0.18
Ice cream, "cornetto"	11.5	3.6	1.0	3.52	0.95	0.06
Ice cream, sandwich type	9.6	3.0	1.1	2.96	0.95	0.11
Muffin, plain	2.6	6.5	9.1	6.35	8.99	0.07
Muffin, with chocolate	7.5	9.4	7.0	9.21	6.84	0.11
Muffin, with fruit	2.1	6.1	8.7	5.95	8.59	0.07
Pastries, plain	5.8	6.8	7.7	6.63	7.47	0.17

Pastries, with chocolate	7.4	7.6	5.5	7.41	5.23	0.20
Pastries, with jam	3.5	4.9	5.5	4.79	5.33	0.13
Pastries, with milk	6.8	4.9	2.7	4.82	2.35	0.28
Pastries, without added sugars	5.3	6.8	5.6	6.70	5.17	0.35
Plum cake	5.1	6.8	8.6	6.62	8.47	0.13
Plum cake, with chocolate	5.9	7.0	7.6	6.87	7.40	0.13
Puff pastry	11.1	8.9	5.2	8.57	4.30	0.85
Snack bar, cereals and chocolate	8.0	4.7	1.3	4.60	1.12	0.09
Snack bar, cereals and nuts	2.9	5.5	3.6	5.39	3.30	0.22
Snack bar, chocolate-coated	13.0	11.5	3.2	11.30	2.86	0.29
Snack roll, "cannolo"	5.4	7.1	4.6	6.88	4.25	0.36
Wafer cone, for ice cream	1.6	1.6	1.3	1.61	1.02	0.22
BREADS						
"Piadina"	1.7	4.2	1.6	4.08	1.50	0.06
"Piadina", wholemeal	1.6	3.5	2.0	3.48	1.93	0.06
Bread, "ciabatta", "baguette", "sfilatino"	0.9	2.4	2.3	2.35	2.26	0.04
Bread, "rosetta", "tartaruga"	0.7	1.2	1.6	1.19	1.51	0.12
Bread, hamburger/ hotdog type	0.8	2.0	1.4	1.95	1.38	0.04
Bread, prepared with oil	1.9	4.5	1.7	4.41	1.55	0.10
Bread, rustic, with seeds	1.1	2.7	4.4	2.66	3.64	0.72
Bread, white, sandwich type	0.7	2.0	2.7	1.99	2.71	0.03
Bread, white, sliced	1.2	2.5	2.4	2.42	2.31	0.05
Bread, wholemeal	1.2	2.7	3.8	2.64	3.48	0.27
Bread, with olives	1.2	4.7	1.5	4.67	1.41	0.05
Breadcrumb	2.2	2.4	1.5	2.37	1.30	0.19
Tortilla wrap	2.1	2.0	2.8	1.96	2.57	0.18
PIZZAS						
"Calzone", frozen	4.1	3.2	1.7	2.91	1.56	0.14
"Focaccia"	1.9	4.9	1.7	4.87	1.68	0.07
Pizza dough, cooked	2.1	4.1	2.2	4.05	2.05	0.15
Pizza, tomato and mozzarella	3.8	3.7	1.1	3.49	0.93	0.15
SAVOURY SNACKS						
"Friselle"	0.5	2.2	0.4	2.17	0.39	0.04
"Taralli"	2.1	11.2	6.2	11.06	5.92	0.30
Breadsticks	2.6	4.8	2.5	4.70	2.47	0.07
Breadsticks, wholemeal	1.6	5.9	6.6	5.84	6.40	0.19
Cheese and cereals snacks	1.4	2.4	1.7	2.24	1.54	0.14
Crackers snacks	3.0	8.1	7.6	7.93	7.40	0.19
Crackers, salted	4.1	4.5	4.9	4.47	4.62	0.23
Crackers, wholemeal	2.8	5.1	4.8	4.98	4.34	0.43
Croutons	3.5	3.7	1.7	3.65	1.47	0.24
Saltines snacks	5.7	4.8	1.6	4.68	1.19	0.39
FLOURS						
Flour, for bread and pizza	0.2	0.2	0.2	0.17	0.18	0.01
Flour, for cakes	0.6	0.3	0.5	0.30	0.42	0.04
Flour, for pasta	0.1	0.1	0.2	0.13	0.17	0.02
Flour, rustic	0.4	0.4	1.0	0.39	0.36	0.66
Flour, unspecified	0.3	0.2	0.2	0.19	0.22	0.02
PASTA DISHES						
"Ravioli", filled with meat	5.0	6.0	2.6	5.50	2.13	0.13
"Ravioli", mixed fillings, fresh	2.7	2.0	0.9	1.81	0.79	0.07

"Ravioli", spinach and ricotta cheese	3.9	2.5	1.1	2.26	0.99	0.11
"Tortellini", filled with meat	3.9	3.5	1.9	3.25	1.71	0.08
Cous cous	0.5	0.6	1.0	0.55	0.85	0.12
Egg pasta, dry	1.5	1.2	0.9	1.10	0.78	0.08
Egg pasta, fresh	1.0	2.2	0.6	2.07	0.50	0.04
Gnocchi	0.1	0.1	0.1	0.07	0.10	0.02
Legume pasta	0.6	0.7	1.3	0.67	1.14	0.19
Pasta, buckwheat	0.7	0.9	0.9	0.85	0.81	0.06
Pasta, corn	0.4	0.3	0.5	0.27	0.44	0.08
Pasta, for broth	0.4	0.5	0.7	0.45	0.64	0.09
Pasta, mixed cereals	0.5	0.5	0.8	0.52	0.73	0.09
Pasta, mixed cereals and legumes	0.6	0.8	1.3	0.74	1.13	0.13
Pasta, rice	0.5	0.6	0.5	0.53	0.48	0.05
Pasta, wholemeal	0.8	0.9	0.9	0.85	0.86	0.05
READY-TO-EAT DISHES						
“Lasagne” with meat	3.1	2.3	0.8	2.03	0.63	0.08
Breaded cheese, frozen	2.6	1.7	0.9	1.54	0.83	0.07
Chicken breast, breaded, frozen	1.8	4.1	5.7	3.94	5.50	0.07
Fish, breaded, frozen	1.2	4.8	4.1	4.39	3.45	0.37
Pasta with pesto sauce	0.8	2.0	1.6	1.98	1.53	0.10
Pasta with tomato sauce	0.4	0.7	0.6	0.64	0.50	0.06
Soup powder, with cereals, mixed	0.5	1.1	1.5	1.03	1.33	0.15

Abbreviations: SFAs, saturated fatty acids; MUFAs, monounsaturated fatty acids; PUFAs polyunsaturated fatty acids.

Table S3. Micronutrient composition per 100 g of gluten free foods from the following categories: cookies, breakfast products, cakes and desserts, sweet snacks, breads, pizzas, savory snacks, flours, pasta dishes, ready-to-eat dishes.

Foods	Fe	Ca	Na	K	P	Zn	Vitamin D	Vitamin E	Ret. eq.	Vitamin B1	Vitamin B2	Niacin	Vitamin B6	Folates	Vitamin C
	mg	mg	mg	mg	mg	mg	µg	mg	µg	mg	mg	mg	mg	µg	mg
BISCUITS															
Biscuits, "canestrelli"	0.8	17	109	110	63	0.50	0.23	3.51	103	0.05	0.05	0.32	0.12	5	0
Biscuits, "cantucci"	1.1	72	194	226	241	1.00	0.41	5.84	64	0.08	0.14	0.88	0.17	15	0
Biscuits, "cantucci", with chocolate	1.3	32	166	159	132	0.68	0.34	2.31	38	0.04	0.08	0.37	0.13	8	0
Biscuits, chocolate-coated	2.1	104	184	254	166	0.65	0.08	1.67	60	0.10	0.15	0.65	0.06	11	1
Biscuits, ladyfinger	1.0	43	201	345	207	1.02	0.79	0.58	81	0.06	0.13	0.40	0.15	18	0
Biscuits, plain	0.7	57	320	251	156	0.66	0.17	3.41	56	0.07	0.09	0.53	0.11	7	0
Biscuits, wholemeal	1.8	53	230	211	182	1.28	0.15	3.39	70	0.18	0.09	1.39	0.17	13	0
Biscuits, with chocolate	1.3	40	146	227	112	0.77	0.16	2.92	57	0.07	0.08	0.51	0.09	8	0
Biscuits, with coconut	1.0	45	132	141	105	0.75	0.22	1.87	126	0.09	0.09	0.93	0.14	7	0
Biscuits, with jam	1.0	34	140	132	82	0.49	0.14	2.47	38	0.06	0.05	0.69	0.11	6	1
Breakfast biscuits	0.9	41	299	290	139	0.64	0.05	3.67	35	0.07	0.07	0.66	0.12	16	0
Filled biscuits	1.5	60	200	227	122	0.72	0.05	4.10	18	0.06	0.08	0.43	0.09	9	0
Tea biscuits	1.1	83	193	143	175	0.61	0.16	2.74	101	0.08	0.11	0.46	0.10	13	0
Wafers, chocolate	1.7	36	82	471	140	1.08	0.00	3.26	6	0.06	0.06	0.46	0.05	17	0
Wafers, chocolate-coated	2.4	157	78	433	188	1.06	0.00	3.73	18	0.09	0.22	0.59	0.13	12	0
Wafers, hazelnut	1.1	89	73	593	151	1.05	0.00	2.84	3	0.07	0.09	0.43	0.10	21	0
Wafers, vanilla	0.4	107	77	426	132	0.69	0.00	0.20	1	0.04	0.12	0.23	0.10	11	0
BREAKFAST PRODUCTS															
Cereal rusks	2.0	27	430	340	139	1.38	0.00	0.32	16	0.18	0.08	1.63	0.28	19	0
Melba toast	0.8	23	666	434	51	0.76	0.00	2.35	0	0.06	0.05	0.76	0.17	33	0
Melba toast, wholemeal	0.9	44	448	332	67	0.65	0.00	2.28	0	0.08	0.07	0.82	0.17	29	0
Muesli	3.2	55	149	380	208	1.73	0.00	2.14	5	0.32	0.17	2.12	0.27	57	1
CAKES AND DESSERTS															
Cake, "colomba"	1.3	38	240	134	136	0.67	0.40	6.64	43	0.05	0.09	0.25	0.09	9	0
Cake, "margherita"	0.5	48	231	166	166	0.51	0.29	3.82	55	0.03	0.07	0.23	0.09	8	2
Cake, "pandoro"	0.8	61	162	163	93	0.64	0.50	0.70	181	0.05	0.13	0.33	0.10	29	0

Cake, "panettone"	1.2	54	154	185	90	0.58	0.52	0.70	143	0.07	0.13	0.49	0.12	37	0
Cake, "panettone", with chocolate	1.4	42	144	130	100	0.67	0.52	0.70	122	0.06	0.12	0.42	0.10	33	0
Cake, chocolate	1.0	33	244	86	164	0.61	0.36	7.27	38	0.04	0.08	0.42	0.11	13	0
Dessert, "tiramisù"	0.8	97	62	197	121	0.68	0.24	0.68	176	0.04	0.16	0.29	0.07	12	1
Sponge cake	0.7	92	60	127	161	0.64	0.53	0.34	54	0.05	0.17	0.14	0.09	9	0
Sweet bread	0.7	29	234	75	50	0.34	0.13	0.93	17	0.05	0.08	0.50	0.14	28	0
Tart, with chocolate and hazelnut	1.1	82	129	158	120	0.73	0.53	1.02	156	0.07	0.16	0.38	0.10	28	0
Tart, with jam	1.0	44	167	165	101	0.50	0.09	3.42	20	0.05	0.06	0.72	0.12	5	1
SWEET SNACKS															
Croissant	1.0	48	258	134	125	0.55	0.18	2.19	23	0.07	0.10	0.67	0.13	33	0
Croissant, with chocolate	1.2	53	249	141	142	0.56	0.09	1.88	25	0.06	0.08	0.62	0.11	30	0
Croissant, with jam	1.0	39	200	106	103	0.44	0.14	1.57	31	0.07	0.10	0.80	0.11	47	1
Ice cream, "cornetto"	0.9	58	61	148	86	0.58	0.01	1.40	21	0.05	0.09	0.38	0.07	7	0
Ice cream, sandwich type	1.1	61	104	164	134	0.76	0.02	1.55	23	0.08	0.10	0.46	0.07	7	0
Muffin, plain	0.6	27	384	131	114	0.56	0.43	6.85	46	0.04	0.08	0.30	0.10	9	0
Muffin, with chocolate	1.6	74	218	226	140	0.73	0.11	5.32	24	0.06	0.08	0.54	0.13	9	0
Muffin, with fruit	0.8	25	205	121	102	0.52	0.29	6.30	33	0.04	0.06	0.48	0.08	7	1
Pastries, plain	1.1	56	251	179	179	0.74	0.60	6.09	104	0.05	0.12	0.29	0.11	14	0
Pastries, with chocolate	1.4	66	195	226	157	0.81	0.39	4.99	44	0.05	0.11	0.30	0.09	12	0
Pastries, with jam	0.8	39	189	147	108	0.48	0.42	4.46	50	0.04	0.09	0.31	0.08	9	3
Pastries, with milk	0.6	70	201	239	140	0.63	0.41	3.08	59	0.04	0.13	0.17	0.07	10	1
Pastries, without added sugars	1.2	37	218	215	134	0.70	0.43	5.70	64	0.05	0.09	0.39	0.13	12	3
Plum cake	0.7	58	312	129	154	0.56	0.41	6.67	48	0.04	0.11	0.23	0.10	9	0
Plum cake, with chocolate	1.1	40	231	139	145	0.67	0.37	5.90	41	0.04	0.09	0.33	0.09	9	0
Puff pastry	0.8	22	386	60	60	0.35	0.00	4.64	0	0.04	0.02	0.50	0.11	5	0
Snack bar, cereals and chocolate	2.6	88	120	268	132	1.01	0.07	1.63	10	0.12	0.20	1.70	0.22	18	0
Snack bar, cereals and nuts	2.0	50	193	303	117	0.90	0.00	4.02	33	0.25	0.32	4.57	0.50	75	0
Snack bar, chocolate-coated	2.1	123	97	280	160	0.93	0.00	6.29	22	0.13	0.18	0.77	0.15	13	0
Snack roll, "cannolo"	1.6	67	78	248	131	1.04	0.00	4.14	4	0.07	0.09	0.83	0.19	11	0

Wafer cone, for ice cream	0.8	24	250	451	102	0.85	0.00	0.98	1	0.06	0.03	0.68	0.22	26	0
BREADS															
"Piadina"	0.6	41	724	132	162	0.53	0.00	1.54	5	0.04	0.03	0.66	0.17	5	0
"Piadina", wholemeal	0.9	35	806	133	156	0.57	0.00	1.66	5	0.09	0.03	0.91	0.16	7	0
Bread, "ciabatta", "baguette", "sfilatino"	1.2	20	502	69	50	0.33	0.00	1.70	2	0.06	0.06	0.65	0.14	36	0
Bread, "rosetta", "tartaruga"	1.1	18	513	136	42	0.37	0.00	1.05	2	0.05	0.04	0.60	0.13	22	0
Bread, hamburger/ hotdog type	0.9	18	640	83	60	0.31	0.00	1.18	1	0.05	0.05	0.72	0.16	31	1
Bread, prepared with oil	0.6	22	476	71	38	0.34	0.00	1.74	1	0.06	0.06	0.74	0.16	38	0
Bread, rustic, with seeds	2.0	49	462	174	117	0.96	0.00	3.05	0	0.13	0.08	1.03	0.19	40	0
Bread, white, sandwich type	1.5	24	513	98	59	0.32	0.00	2.00	0	0.05	0.04	0.54	0.11	19	0
Bread, white, sliced	1.1	21	547	98	49	0.39	0.00	1.78	1	0.06	0.06	0.81	0.15	30	0
Bread, wholemeal	1.5	24	586	104	80	0.64	0.00	2.78	1	0.11	0.06	1.18	0.19	27	0
Bread, with olives	1.6	61	590	193	69	0.53	0.00	1.38	4	0.06	0.10	0.69	0.13	35	2
Breadcrumb	1.2	26	461	149	83	0.67	0.01	1.37	16	0.12	0.08	1.02	0.16	27	0
Tortilla wrap	1.3	34	915	163	123	0.69	0.00	1.93	2	0.06	0.03	0.83	0.15	9	1
PIZZAS															
"Calzone", frozen	1.8	85	300	265	119	1.09	0.20	2.75	282	0.09	0.14	0.81	0.21	46	6
"Focaccia"	1.1	33	956	84	45	0.31	0.00	1.76	3	0.06	0.08	0.62	0.14	38	0
Pizza dough, cooked	0.9	32	722	190	108	0.48	0.00	1.88	1	0.06	0.07	0.69	0.15	36	0
Pizza, tomato and mozzarella	1.0	65	493	185	137	0.96	0.03	1.64	133	0.07	0.11	0.88	0.17	34	6
SAVOURY SNACKS															
"Friselle"	0.7	96	804	338	165	0.75	0.01	0.65	11	0.09	0.14	0.78	0.17	26	0
"Taralli"	0.7	30	1081	326	96	0.61	0.00	4.74	4	0.04	0.03	0.56	0.17	13	0
Breadsticks	0.8	32	880	278	71	0.64	0.01	2.41	4	0.08	0.06	0.72	0.20	35	0
Breadsticks, wholemeal	0.7	23	935	194	78	0.60	0.00	4.85	1	0.08	0.05	0.77	0.18	29	0
Cheese and cereals snacks	1.0	51	776	141	118	0.88	0.01	0.98	34	0.11	0.09	1.30	0.20	10	0
Crackers snacks	0.7	31	861	301	116	0.51	0.05	5.72	11	0.06	0.04	0.43	0.15	13	0
Crackers, salted	0.9	33	768	215	117	0.54	0.02	3.84	7	0.09	0.06	0.82	0.18	25	0
Crackers, wholemeal	1.9	42	621	337	160	1.32	0.05	3.39	9	0.14	0.08	1.67	0.20	37	0

Croutons	0.8	28	788	75	37	0.28	0.01	2.00	5	0.08	0.08	0.60	0.16	38	0
Saltines snacks	0.6	15	1652	225	57	0.52	0.07	3.01	8	0.06	0.07	0.73	0.21	37	0
FLOURS															
Flour, for bread and pizza	0.7	31	382	232	58	0.54	0.00	0.06	0	0.08	0.05	0.53	0.20	14	0
Flour, for cakes	0.9	34	50	205	92	0.58	0.00	0.14	5	0.09	0.05	0.49	0.17	16	0
Flour, for pasta	0.6	47	201	137	61	0.46	0.00	0.04	7	0.10	0.08	0.58	0.20	4	0
Flour, rustic	1.4	29	450	150	121	1.12	0.00	0.20	0	0.20	0.04	1.35	0.21	18	0
Flour, unspecified	0.5	28	131	216	41	0.44	0.00	0.07	1	0.07	0.04	0.48	0.20	8	0
PASTA DISHES															
"Ravioli", filled with meat	1.7	110	292	209	198	1.72	0.83	1.71	107	0.12	0.14	1.37	0.27	24	0
"Ravioli", mixed fillings, fresh	0.9	94	234	156	117	0.64	0.38	0.81	114	0.07	0.09	0.41	0.09	22	2
"Ravioli", spinach and ricotta cheese	1.3	200	557	310	205	1.16	0.36	1.30	164	0.11	0.23	0.70	0.18	35	6
"Tortellini", filled with meat	1.4	74	858	257	173	1.30	0.68	1.52	98	0.17	0.16	1.51	0.24	27	1
Cous cous	2.4	30	2	295	160	1.75	0.00	0.35	37	0.35	0.10	2.16	0.26	41	1
Egg pasta, dry	1.0	23	197	151	92	0.73	0.39	0.29	76	0.14	0.11	0.76	0.18	14	0
Egg pasta, fresh	1.3	27	84	402	114	1.03	0.40	0.76	56	0.14	0.11	0.75	0.13	25	1
Gnocchi	0.7	12	420	559	66	0.50	0.00	0.12	14	0.11	0.05	2.28	0.41	34	13
Legume pasta	5.2	101	4	939	323	2.99	0.00	0.91	14	0.48	0.18	1.89	0.59	112	3
Pasta, buckwheat	2.0	12	0	220	150	2.60	0.00	0.00	0	0.28	0.07	2.80	0.40	54	0
Pasta, corn	1.8	7	6	132	99	1.00	0.00	0.01	64	0.34	0.10	1.89	0.01	0	0
Pasta, for broth	1.4	11	9	209	112	1.05	0.00	0.15	26	0.23	0.07	1.76	0.22	16	0
Pasta, mixed cereals	1.5	12	1	173	144	1.24	0.00	0.28	31	0.28	0.09	2.10	0.22	16	0
Pasta, mixed cereals and legumes	2.8	50	6	506	180	1.76	0.00	0.55	16	0.29	0.09	1.59	0.37	57	1
Pasta, rice	0.5	8	16	120	115	0.90	0.00	0.22	0	0.11	0.04	1.78	0.43	11	0
Pasta, wholemeal	1.4	10	23	225	270	1.66	0.00	0.66	6	0.53	0.07	4.64	0.43	42	0
READY-TO-EAT DISHES															
"Lasagne" with meat	0.7	57	248	166	89	0.98	0.25	1.39	209	0.08	0.11	0.98	0.19	20	6
Breaded cheese, frozen	0.7	206	480	249	187	1.22	0.21	1.44	179	0.11	0.23	0.71	0.21	23	4
Chicken breast, breaded, frozen	0.7	17	498	268	160	0.73	0.13	5.04	5	0.09	0.13	4.42	0.31	14	0

Fish, breaded, frozen	0.7	22	376	254	139	1.32	0.52	3.60	19	0.08	0.07	1.41	0.19	10	0
Pasta with pesto sauce	1.4	62	141	141	63	0.55	0.01	1.73	32	0.06	0.04	0.89	0.15	5	0
Pasta with tomato sauce	1.4	29	272	204	121	1.00	0.00	0.96	181	0.16	0.08	1.98	0.38	25	11
Soup powder, with cereals, mixed	4.0	64	13	612	296	2.74	0.00	0.91	16	0.39	0.14	2.25	0.36	74	2

Abbreviations: Ret. eq., retinol equivalent
